# Maslinic Acid Enhances Docetaxel Response in Human Docetaxel-Resistant Triple Negative Breast Carcinoma MDA-MB-231 Cells *via* Regulating MELK-FoxM1-ABCB1 Signaling Cascade

**DOI:** 10.3389/fphar.2020.00835

**Published:** 2020-06-09

**Authors:** Ke Wang, Xue Zhu, Yongxiang Yin

**Affiliations:** ^1^NHC Key Laboratory of Nuclear Medicine, Jiangsu Key Laboratory of Molecular Nuclear Medicine, Jiangsu Institute of Nuclear Medicine, Wuxi, China; ^2^Department of Pathology, the Afﬁliated Maternity and Children Health Hospital of Nanjing Medical University, Wuxi, China

**Keywords:** triple negative breast cancer, docetaxel resistance, maslinic acid, MELK-FoxM1-ABCB1 signaling cascade, MDA-MB-231

## Abstract

Docetaxel (DOC) is the most important chemotherapeutic drug for the treatment of triple negative breast cancer (TNBC); however, acquired drug resistance upon the long-term treatment limits its therapeutic effect. Maslinic acid (MA), a natural triterpene from *Olea europaea L*., attracts increasing interest in recent years because of its promising anti-cancer activity, but the reversal effect of MA on drug resistance in cancer therapy is rarely explored. In this study, the combined effect of DOC and MA on human docetaxel-resistant triple negative breast carcinoma MDA-MB-231 (MDA-MB-231/DOC) cells was investigated. The enhanced effect of MA on DOC cytotoxicity and DOC accumulation was assessed by MTT (3-(4,5-dimethylthiazol-2-yl)-2,5-diphenyltetrazolium bromide) assay and HPLC (high performance liquid chromatography) analysis in MDA-MB-231/DOC cells. Western blot, co-immunoprecipitation assay, luciferase reporter assay, and chromatin immunoprecipitation (ChIP) assay were performed for exploring the underlying mechanisms. Our data indicated that the co-treatment of MA could dose-dependently enhance DOC sensitivity and cellular DOC accumulation in MDA-MB-231/DOC cells. Moreover, MELK-FoxM1-ABCB1 signaling cascade was confirmed to contribute to DOC resistance in MDA-MB-231/DOC cells. In such process, MA directly suppressed expressions and interaction of MELK and FoxM1 as well as the transcriptional activity of FoxM1, and thus reducing the expression of ABCB1. Overall, our study suggests that the combined use of DOC and MA may be helpful for overcoming DOC resistance in human TNBC therapy.

## Introduction

Though the improvement of surgical technique and the development of adjuvant chemotherapeutic regimens over the last 30 years, the survival rate of triple negative breast cancer (TNBC) is still low and which is the leading cause of cancer-related women death (Collignon et al., 2016; Lebert et al., 2018). Chemotherapy is one of main management tools for TNBC control (Bianchini et al., 2016). Among generation chemotherapy regimen for TNBC, docetaxel (DOC) is widely used as one of first-line chemotherapeutic drugs (Sharma, 2016). Resistance to DOC is the main reason for the failure of chemotherapy regimens (O’Reilly et al., 2015), thus, investigation of novel therapeutic strategy that may allow reversion of docetaxel resistance is important for improving chemotherapeutic effect in TNBC.

MELK (Maternal embryonic leucine-zipper kinase)/FoxM1(Forkhead box M1) signaling is estimated to be up-regulated in various cancers and associated with tumor growth, metastasis, angiogenesis and chemoresistance (Joshi et al., 2013; Kim et al., 2015). MELK is a serine/threonine kinase belonging to the family of AMPK/snf1 protein kinases and acts as an important regulator in cytokinesis, cell proliferation, cell cycle and cell apoptosis (Wang et al., 2014). The oncogenic properties of MELK make it to be a key functional regulator of drug resistance in several lines of cancers. Marisa et al. have reported that the expression of MELK is increased in mammary stem cells from undifferentiated cancers, which is associated with poor prognosis and potentially mediates treatment resistance (Simon et al., 2017). MELK forms a protein complex with the oncogene FoxM1, a typical proliferation-associated transcription factor involved in tumorigenesis. FoxM1 phosphorylation regulated by MELK facilitates FoxM1 transcriptional activity and induces the expression of various mitotic regulators (Joshi et al., 2013; Joshi et al., 2014). Park et al. have reported that FoxM1 is a poor prognostic factor of TNBC and mediates its resistance to docetaxel (Park et al., 2012). Thus, targeting MELK-FoxM1 signaling may provide a novel strategy to benefit patients of TNBC undergoing chemotherapy treatment.

Triterpenoids are a diverse group of natural products composed of three terpene units and exhibit cytotoxicity against a variety of tumor cells (Liby et al., 2007). Maslinic acid (MA) is an ursane type triterpene and widely distributed in food and plants. The anti-cancer effect of MA has been confirmed in multiple types of human cancers including TNBC (Reyes-Zurita et al., 2009; Li et al., 2010; Lin et al., 2014; Parikh et al., 2014). Yu et al. have reported that MA can enhance the anti-tumor effect of gemcitabine in gallbladder cancer cancer cells by inhibiting transcription factor nuclear factor-kappa B (Yu et al., 2015). Villar et al. have reported that MA sensitizes soft tissue sarcoma cells to DOC by inhibiting the multidrug resistance protein MRP-1 (Villar et al., 2014). In this experiment, the reversal effect of MA on DOC resistance in human docetaxel-resistant triple negative breast carcinoma MDA-MB-231 cells, and the role of MELK-FoxM1 signaling cascade in MA’s effect was explored.

## Materials and Methods

### Materials

Maslinic acid (purity > 98%), docetaxel, DMSO (dimethyl sulfoxide) and MTT (3-(4,5-dimethylthiazol-2-yl)-2,5-diphenyltetrazolium bromide) were obtained from Sigma-Aldrich (MO, USA). The apoptosis kit was obtained from BD Biosciences (CA, USA). Lipofectamine 2000 was obtained from Invitrogen (CA, USA). Antibodies in this experiment were obtained from the companies of Santa Cruz Biotechnology (CA, USA) and Abcam (MA, USA). The antibodies used in this study were shown as following: ABCB1 (sc-55510, Santa Cruz, 1:1,000), MELK (ab273015, Abcam, 1:1,000), FoxM1 (ab207298, Abcam, 1:1,000), GAPDH (sc-365062, Santa Cruz, 1:500). The other materials were obtained from Beyotime (Nantong, China).

### Cell Culture and Transfection

The human triple negative breast carcinoma MDA-MB-231 cells were obtained from American Type Culture Collection (ATCC, VA, USA). The docetaxel-resistant MDA-MB-231 cells (MDA-MB-231/DOC) were established as previously reported (Dey et al., 2017). Initially, cells were treated with DOC (20 nM) for 48 h and then kept in fresh medium for 72 h. Live cells were again treated with 40 nM DOC for 48 h and then kept in fresh medium for 72 h. In this way the dose of DOC was increased from 20 nM to 1,000 nM. At the end of one year, cells became resistant (MDA-MB-231/DOC). Cells were cultured in the medium of DMEM (dulbecco’s modified eagle medium), which were added with fetal bovine serum (FBS, 10%) and penicillin-streptomycin (1%). Cells were maintained at 37°C in a humidified atmosphere containing 5% CO_2_. For the measurement of MA activity, cells were pre-treated by the expression plasmids (FoxM1 and MELK) transfection using Lipofectamine 2000.

### Measurement of Cell Viability

For measurement of cell viability, MTT assay was conducted according to the previous study (van Meerloo et al., 2011). Following treatment, MTT solution was added each well and the incubated for 2 h at 37°C. The culture medium was removed and 100 μl DMSO was added to dissolve the formazan crystals. The absorbance of the cell suspension was measured at 570 nm using a microplate reader (Bio-Rad, CA, USA).

### Measurement of Cellular Drug Accumulation

For measurement of cellular drug accumulation, high performance liquid chromatography (HPLC) analysis was conducted according to the previous study (Yan et al., 2019). Cells were incubated with 1 μg/ml DOC at 37 °C for 2 h, and sonicated in 0.2% SDS (sodium dodecyl sulfate)/PBS (phosphate buffered saline) for 15 min. Following treatment, supernatant was collected and concentration of cellular DOC was assessed by HPLC using a diamond C18 reversed-phase column (4.6 mm × 250 mm). The mobile phase was acetonitrile and water (55:45, v/v) with potassium dihydrogen phosphate (0.06 M), and the ﬂow rate was 0.8 ml/min and pH was adjusted to 5.0.

### Western Blot Analysis

For measurement of protein expression, western blot analysis was conducted according to the previous study (Wang et al., 2016b). Following treatment, cells lysates were resolved by SDS-PAGE, electrophoretically transferred onto polyvinylidene fluoride (PVDF) membrane, blotted with the primary and secondary antibodies and detected by ECL detection kit (Beyotime, Nantong, China).

### Co-Immunoprecipitation Analysis

For measurement of protein interaction, co-immunoprecipitation analysis was used as previously mentioned (Wang et al., 2016b). Following treatment, cell exacts were incubated with antibody at 4°C for 24 h, followed by incubation with protein A-Sepharose 4B or beads at 4°C for 4 h. Then, the input, immunoprecipitate and flow through fractions were analyzed by western blot analysis.

### Luciferase Reporter Assay

For measurement of the transcriptional activity of FoxM1, luciferase report assay was conducted according to the previous study (Smale, 2010). Following FoxM1-luc reporter vector (containing FoxM1 proximal promoter) transfection, cells were treated with agent and luciferase report assay was performed 48 h later. Then the relative luciferase activity was analyzed. For the dual-luciferase assay, the human ABCB1 gene promoter was sub-cloned into pGL3 vector (wild-type:5′-TTTGTTTGTTTT-3′ or mutant: 5′-TCCATCCAGGGT-3′) (Promega, MA, USA) in HEK293T cells. Cells were co-transfected with ABCB1 promoter vector, pcDNA-FoxM1 and internal control plasmid, and luciferase assays were performed 48 h after transfection using the Fireﬂy/Renilla Dual Luciferase Reporter Assay System according to the manufacturer’s instructions (Promega, MA, USA).

### Chromatin Immunoprecipitation (ChIP) Assay

Chromatin immunoprecipitation assay was performed with the ChIP assay kit (Cell Signaling, MA, USA). Cells were cross-linked and lysed. Sheared chromatin DNA mixture (normalized inputs) was collected and incubated with FoxM1 antibody overnight at 4°C. The resulting precipitated DNA samples were analyzed by RT-PCR. The PCR products were resolved electrophoretically on a 2% agarose gel and visualized by ethidium bromide staining.

### Statistical Analysis

GraphPad Prism 5.0 (GraphPad, CA, USA) was used for biostatistical analyses. All data were presented as means ± SD with three independent experiments, and triplicate repeats were included in each experiment. Student’s *t*-test was performed for comparison of means between the two groups. *p* < 0.05 was considered to be significant difference.

## Results

### MA Enhances the Inhibitory Effect of DOC on Cell Viability in MDA-MB-231/DOC Cells

**Figure 1:**
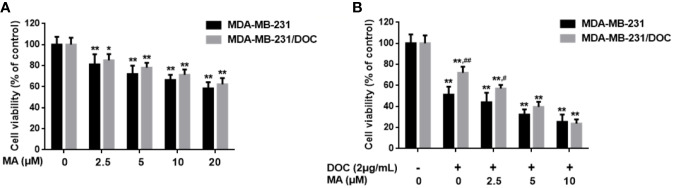
The combined effect of docetaxel (DOC) and maslinic acid (MA) on cell viability in MDA-MB-231/DOC cells. **(A)** MDA-MB-231 and MDA-MB-231/DOC cells were incubated with MA alone for 24 h, and cell viability was evaluated by MTT assay. ^*^p < 0.05 *vs.* control, ^**^p < 0.01 *vs.* control. **(B)** MDA-MB-231 and MDA-MB-231/DOC cells were incubated with DOC alone or DOC combined with MA for 24 h, and cell viability was evaluated by MTT assay. ^**^p < 0.01 *vs.* control, ^#^*p <* 0.05, ^##^*p <* 0.01 *vs.* MDA-MB-231 group. All data were presented as means ± SD with three independent experiments, and triplicate repeats were included in each experiment.

MTT assay was used to investigate the combined effect of DOC and MA on cell viability in MDA-MB-231 and MDA-MB-231/DOC cells. First, the results indicated that DOC (2 μg/ml) treatment resulted in 48.83% reduction of cell viability for MDA-MB-231 while that for MDA-MB-231/DOC was 34.76%. However, the co-treatment of MA significantly increased the sensitivity of MDA-MB-231/DOC cells to docetaxel, which was in a dose-dependent manner (**Figure 1**).

### MA Enhances the Cellular Drug Accumulation of DOC in MDA-MB-231/DOC Cells

**Figure 2:**
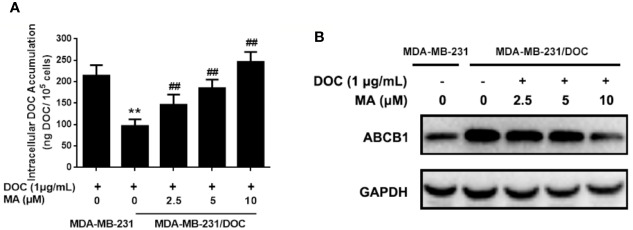
The effect of maslinic acid (MA) on cellular docetaxel (DOC) accumulation in MDA-MB-231/DOC cells. MDA-MB-231 and MDA-MB-231/DOC cells were incubated with DOC alone or DOC combined with MA for 24 h. **(A)** Cell lysates were collected for testing of cellular DOC concentrations using HPLC. **(B)** The expression of drug efflux protein ABCB1 was assessed by western blot analysis. ^**^p < 0.01 *vs.* MDA-MB-231 with DOC treatment, ^##^*p <* 0.01 *vs.* MDA-MB-231/DOC with DOC treatment. All data were presented as means ± SD with three independent experiments, and triplicate repeats were included in each experiment.

Cellular DOC accumulation analysis was used to investigate the effect of MA on DOC efflux in MDA-MB-231/DOC cells. First, the results indicated that cellular DOC concentration for MDA-MB-231/DOC cells treated with DOC was significantly lower than that for MDA-MB-231 cells. However, the co-treatment of MA resulted in dose-dependently enhanced cellular DOC concentration in MDA-MB-231/DOC cells (**Figure 2A**). Then, the change of drug efflux-related protein expression upon the indicated treatment was analyzed. As shown in **Figure 2B**, the expression of ABCB1 was higher in MDA-MB-231/DOC cells compared to those in MDA-MB-231 cells; however, the co-treatment of MA significantly reduced such effect in MDA-MB-231/DOC cells.

### MA Inhibits the Activation of MELK-FoxM1 Signaling in MDA-MB-231/DOC Cells

**Figure 3:**
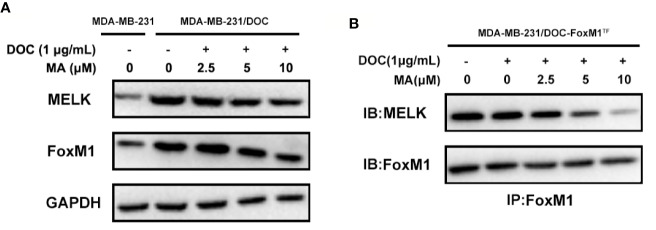
The combined effect of docetaxel (DOC) and maslinic acid (MA) on the expressions and interaction of maternal embryonic leucine-zipper kinase (MELK) and FoxM1 in MDA-MB-231/DOC cells. **(A)** MDA-MB-231 and MDA-MB-231/DOC cells were incubated with or without the indicated drugs for 24 h, and western blot analysis was used to evaluate the expression levels of MELK and FoxM1. **(B)** MDA-MB-231/DOC-FoxM1^TF^ cells were incubated with or without the indicated drugs for 24 h, and co-immunoprecipitation analysis was used to evaluate the interaction of MELK and FoxM1. Every figure was representative of three independent experiments.

**Figure 4:**
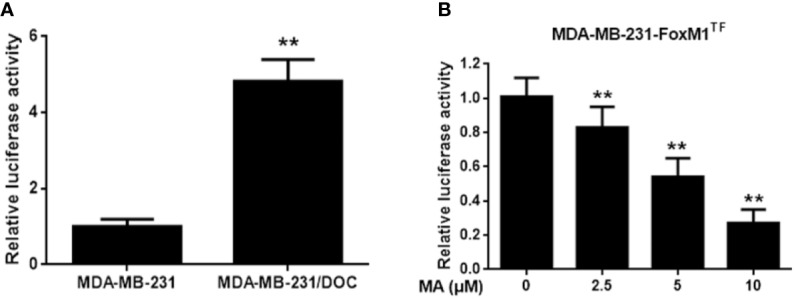
The effect of maslinic acid (MA) on the transcriptional activity of FoxM1 in MDA-MB-231/DOC cells. **(A)** MDA-MB-231 and MDA-MB-231/DOC cells were transfected with FoxM1-luc reporter vector, and the transcriptional activity of FoxM1 was assessed. ^**^p < 0.01 *vs.* MDA-MB-231 group. **(B)** MDA-MB-231/DOC-FoxM1^TF^ cells transfected with FoxM1-luc reporter vector were treated with or without MA for 24 h, and luciferase report assay was conducted to evaluate the transcriptional activity of FoxM1. ^**^p < 0.01 *vs.* control. All data were presented as means ± SD with three independent experiments, and triplicate repeats were included in each experiment.

To investigate the mechanism responsible for MA’s potentiated effect, the change of MELK-FoxM1 signaling, previously reported to be the target of ursane type triterpene, was further analyzed (Wang et al., 2018). First, the expressions of MELK and FoxM1 were up-regulated in MDA-MB-231/DOC cells compared to those in MDA-MB-231 cells; however, which were significantly suppressed by the co-treatment of MA (**Figure 3A**). Then, MA’s effect on the interaction of MELK and FoxM1 was assessed using MDA-MB-231/DOC cells with FoxM1 transfection (MDA-MB-231/DOC-FoxM1^TF^) and the results showed that MA treatment could attenuate such interaction (**Figure 3B**). Finally, the change of the transcriptional activity of FoxM1 upon MA treatment was further investigated. The results showed the transcriptional activity of FoxM1 was up-regulated in MDA-MB-231/DOC cells compared to that of MDA-MB-231 cells (**Figure 4A**), and the treatment of MA could dose-dependently reduce the transcriptional activity of FoxM1 in MDA-MB-231/DOC-FoxM1^TF^ cells (**Figure 4B**). The data indicated MA exerted its activity through suppressing the expressions and interaction of MELK and FoxM1 as well as the transcriptional activity of FoxM1.

### Restoration of MELK-FoxM1 Signaling Reverses MA’s Effect on Cellular DOC Accumulation

**Figure 5:**
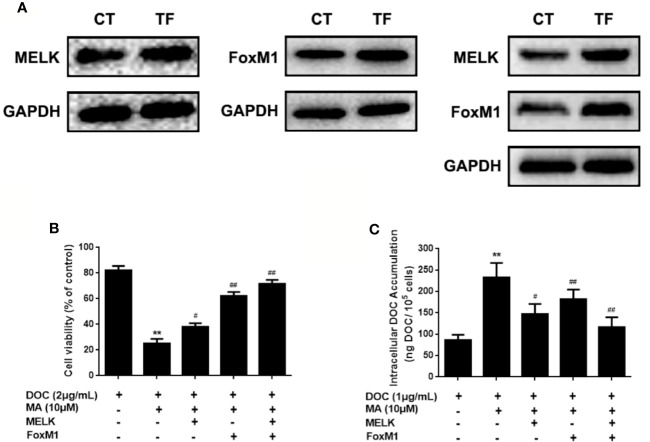
The contribution of MELK-FoxM1 signaling to the potentiated effect of maslinic acid (MA) on cellular docetaxel (DOC) accumulation in MDA-MB-231/DOC cells. MDA-MB-231/DOC cells transfected with maternal embryonic leucine-zipper kinase (MELK) alone, FoxM1 alone or MELK+FoxM1 were treated with DOC alone or DOC combine d MA for 24 h. **(A)** The expressions of MELK and FoxM1 in cells with MELK or FoxM1 transfection alone and MELK and FoxM1 co-transfection were assessed by western blot analysis. **(B)** Cell viability was assessed by MTT assay. **(C)** Cellular DOC accumulation was evaluated by high performance liquid chromatography (HPLC) analysis. ^**^p < 0.01 *vs.* treatment of DOC alone, ^#^*p <* 0.05, ^##^*p <* 0.01 *vs.*co-treatment of DOC and MA. All data were presented as means ± SD with three independent experiments, and triplicate repeats were included in each experiment. CT, control; TF, transfection.

To investigate the contribution of MELK-FoxM1 signaling to MA’s potentiated effect, cells with MELK transfection, FoxM1 transfection or MELK and FoxM1 co-transfection were established (**Figure 5A**). As shown in **Figures 5B, C**, MELK and FoxM1 co-transfection, rather than MELK alone or FoxM1 alone, significantly resulted in the enhanced cell viability and the reduced cellular drug accumulation in DOC and MA co-treated MDA-MB-231/DOC cells.

### Restoration of MELK-FoxM1 Signaling Reverses MA’s Inhibitor Effect on ABCB1 Expression

**Figure 6:**
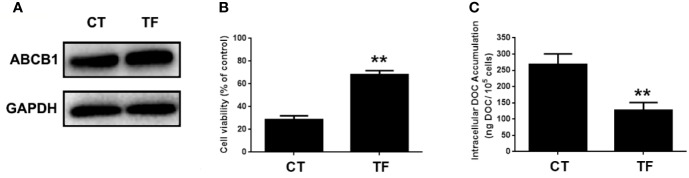
The involvement of ABCB1 in the potentiated effect of maslinic acid (MA) on cellular docetaxel (DOC) accumulation in MDA-MB-231/DOC cells. MDA-MB-231/DOC cells with or without ABCB1 transfection were treated with DOC combined MA for 24 h. **(A)** The expression of ABCB1 in cells transfected with or without ABCB1 expression vector. **(B)** Cell viability was assessed by MTT assay. **(C)** Cellular DOC accumulation was evaluated by high performance liquid chromatography (HPLC) analysis. ^**^p < 0.01 *vs.* CT. All data were presented as means ± SD with three independent experiments, and triplicate repeats were included in each experiment. CT, control; TF, transfection.

**Figure 7:**
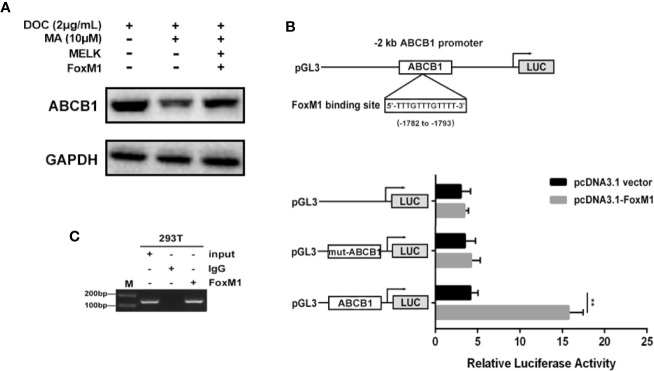
The association of ABCB1 expression and MELK-FoxM1 signaling cascade. **(A)** The expression of ABCB1 in MDA-MB-231/DOC cells with indicated treatment. **(B)** The predicted position of FoxM1 binding site in -2 kb *ABCB1* promoter, and verified by dual-luciferase reporter assay in HEK293T cells. ^**^p < 0.01 *vs.* pcDNA3.1 group. **(C)** Direct binding of FoxM1 to ABCB1 promoter region was determined using ChIP assay. All data were presented as means ± SD with three independent experiments, and triplicate repeats were included in each experiment.

**Figure 8:**
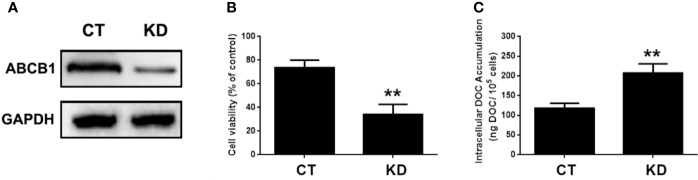
The involvement of ABCB1 in the potentiated effect of MELK-FoxM1 signaling in MDA-MB-231/DOC cells. MDA-MB-231/DOC cells with maternal embryonic leucine-zipper kinase (MELK) and FoxM1 co-transfection were transfected with or without ABCC1 siRNA and then treated with docetaxel (DOC) combined MA for 24 h. **(A)** The expression of ABCB1 in cells with ABCB1 knockdown was assessed by western blot analysis. **(B)** Cell viability was assessed by MTT assay. **(C)** Cellular DOC accumulation was evaluated by high performance liquid chromatography (HPLC) analysis. ^**^p < 0.01 *vs.* CT. All data were presented as means ± SD with three independent experiments, and triplicate repeats were included in each experiment. CT, control; KD, knockdown.

ABCB1 was previously reported to contribute to DOC resistance in various tumor cells. In this experiment, ABCB1 was found to be up-regulated in MDA-MB-231/DOC cells compared to MDA-MB-231 cells, and which was significantly down-regulated upon MA co-treatment. However, the regulatory mechanism of ABCB1 in MA’s potentiated effect was not clear. First, MDA-MB-231/DOC cells with ABCB1 transfection were treated with DOC combined with MA and the results showed the increased cell viability and reduced cellular drug accumulation (**Figure 6**). Then, the association of ABCB1 expression and MELK-FoxM1 signaling cascade was analyzed. The results showed MELK and FoxM1 co-transfection restored the expression of ABCB1 in MDA-MB-231/DOC cells with DOC combine d MA treatment. Moreover, luciferase reporter assay and ChIP assay showed ABCB1 was the target gene of FoxM1, and -1,782 to -1,793 region of ABCB1 promoter was the specific binding site of FoxM1 (**Figure 7**). In addition, ABCB1 knockdown attenuated the effect of MELK and FoxM1 co-transfection on cell viability and cellular drug accumulation (**Figure 8**). The data indicated ABCB1 was regulated by MELK-FoxM1 signaling cascade in MDA-MB-231/DOC cells and played an important role in MA’s potentiated effect.

## Discussion

Maslinic acid (MA) is a natural triterpene and exhibits the anti-tumor properties by regulating cell growth, apoptosis and metabolism. Some of the triterpenes have already passed the clinical trial for cancer treatment, which induce the progression of tumor cell death by targeting multiple cancer-specific targets by the proteasome, B cell lymphoma 2 (Bcl-2), NF-κB, STAT3, TNF, angiogenesis, PI3K/Akt/mTOR, and (TLR) and improve the cytotoxic action of anticancer chemotherapy by inhibiting the function of the MDR-efflux proteins such as MDR1 (Molnar et al., 2006; Gill et al., 2016). Existing evidence has revealed that MA exerts therapeutic effect on a wide variety of human solid tumors, including hepatocellular carcinoma (Ku et al., 2015), colorectal carcinoma (Yoo et al., 2015), gastric carcinoma (Lee et al., 2015), and breast carcinoma (Alam and Khan, 2017). Moreover, MA has been shown to potentiate the anti-tumor activity of conventional chemotherapeutic drugs or reverse chemoresistance of cancer cells to conventional chemotherapeutic drugs (Yu et al., 2015). In this study, we found that MA co-treatment could significantly reverse DOC resistance in human docetaxel-resistant breast carcinoma MDA-MB-231 cells *via* enhancing cellular DOC accumulation.

**Figure 9:**
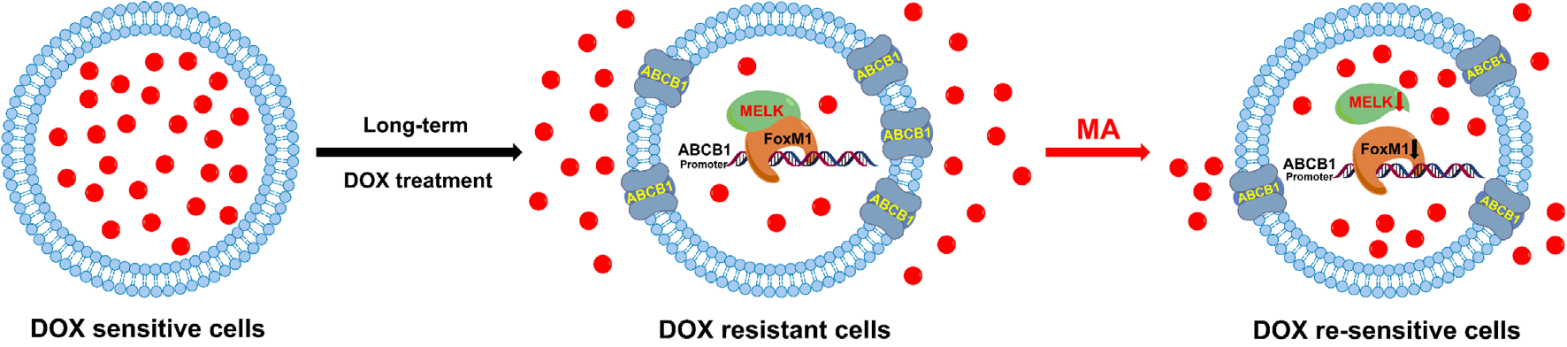
Diagram of the proposed mechanism of MA on DOX response in MDA-MB-231/DOC cells.

Docetaxel is one of the most important chemotherapeutic drugs and exerts its activity *via* enhancement and stabilization of microtubule, leading to an arrest of the cell cycle at the G2/M phase (Kenmotsu and Tanigawara, 2015). Despite initial sensitivity to DOC, most cancer cells will eventually develop resistance (Galletti et al., 2007). Several mechanisms responsible for DOC resistance have been described, which include decrease of cell death activation, drug influx/efflux modifications, or autocrine survival signaling (Wang et al., 2014). Among which, reduced intracellular drug concentration through alteration of multidrug resistant (MDR) gene such as ABCB1 is an important mechanism associated with the acquired resistance to DOC in several tumors (Zhu et al., 2013). FoxM1 (forkhead box protein M1) is a critical proliferation-associated transcription factor, FoxM1-mediated activation of survival pathways following DOC treatment may be an important factor in mediating acquired resistance to DOC (Park et al., 2012; Wang et al., 2016a; Yuan et al., 2018). Li et al. have reported that FoxM1 directly targets and up-regulates the microtubule-destabilizing protein Stathmin, and then prevents the tubulin polymerization, eventually mediates the resistance to DOC-induced apoptosis in gastric cancer cells (Li et al., 2014). Mechanistically, the activity of FoxM1 is regulated by MELK kinase in various cancer cells; however, the contribution of MELK-FoxM1 signaling in the process of DOC resistance acquisition in TNBC is never deeply investigated. In this study, by established the *in vitro* model, we found the expressions and interaction of MELK and FoxM1 as well as the transcriptional activity of FoxM1 were up-regulated and associated with the decreased drug sensitivity and cellular drug accumulation in MDA-MB-231/DOC cells. Thus, the MELK-FoxM1 signaling might be an important contributor in the development of DOC resistance in *in vitro* model and the strategy for the treatment of TNBC cancer could be the combination of DOC-based chemotherapy with agents targeting MELK-FoxM1 signaling cascade. In the reported and our previous studies, triterpenoid compounds such as ursolic acid (UA) and corosolic acid (CA) were estimated to be the effective inhibitor of FoxM1, which exhibited cytotoxic effect on cell viability and inductive effect on G2/M arrest and apoptosis *via* inhibition of the expression levels of MELK and FoxM1 as well as the transcriptional activity of FoxM1 driven by itself or MELK (Wang et al., 2012; Wang et al., 2018). In this experiment, we found MA, the analog of UA and CA, could restore DOC response in MDA-MB-231/DOC cells by directly suppressing the expressions and interaction of MELK and FoxM1 as well as the transcriptional activity of FoxM1, thus reducing the expression of drug efflux-related protein. In addition, further investigation revealed that ABCB1 was the direct target of FoxM1, and -1,782 to -1,793 region of ABCB1 promoter was the specific binding site of FoxM1. ABCB1 knockdown attenuated the effect of MELK and FoxM1 co-transfection on cell viability and cellular drug accumulation. Therefore, the data indicated that MA exerted its activity against DOC resistance mainly through regulating MELK-FoxM1-ABCB1 signaling cascade, and thus enhancing cellular DOC accumulation and reducing cell viability (**Figure 9**).

In conclusion, combining the results of previous studies with those of our study, we confirmed that MELK-FoxM1-ABCB1 signaling cascade was a key target against DOC resistance in TNBC and provided novel insights into the prospecting application of MA or its derivatives as adjuvant chemotherapeutic agent in the treatment of such disease.

## Data Availability Statement

The raw data supporting the conclusions of this article will be made available by the authors, without undue reservation.

## Author Contributions

KW and YY designed experiments. KW and XZ carried out experiments. KW analyzed experimental results. KW wrote the manuscript. YY finished the final version approval.

## Funding

This work was supported by the grants from the Young Talent’s Subsidy Project in Science and Education of the Department of Public Health of Jiangsu Province (No. QNRC2016627), the Six talent peaks project in Jiangsu Province (No. WSW-047), Six-one Scientific Research Project (No. LGY2019087), and the Innovation Capacity Development Plan of Jiangsu Province (No. BM2018023).

## Conflict of Interest

The authors declare that the research was conducted in the absence of any commercial or financial relationships that could be construed as a potential conflict of interest.
